# Inguinal lymph node metastases from rectal adenocarcinoma: a systematic review

**DOI:** 10.1007/s10151-023-02826-x

**Published:** 2023-05-26

**Authors:** James Wyatt, Simon G. Powell, Salma Ahmed, James Arthur, Kiran Altaf, Shakil Ahmed, Muhammad Ahsan Javed

**Affiliations:** 1 Department of Colorectal Surgery, Liverpool University Hospitals NHS Foundation Trust, Liverpool, L7 8XP UK; 2 Institute of Life Course and Medical Sciences, University of Liverpool, Liverpool, L1 8JX UK

**Keywords:** Rectal cancer, Inguinal lymph nodes, Lymphadenectomy, Chemoradiotherapy, Survival

## Abstract

**Purpose:**

Inguinal lymph nodes are a rare but recognised site of metastasis in rectal adenocarcinoma. No guideline or consensus exists for the management of such cases. This review aims to provide a contemporary and comprehensive analysis of the published literature to aid clinical decision-making.

**Methods:**

Systematic searches were performed using the PubMed, Embase, MEDLINE and Scopus and Cochrane CENTRAL Library databases from inception till December 2022. All studies reporting on the presentation, prognosis or management of patients with inguinal lymph node metastases (ILNM) were included. Pooled proportion meta-analyses were completed when possible and descriptive synthesis was utilised for the remaining outcomes. The Joanna Briggs Institute tool for case series was used to assess the risk of bias.

**Results:**

Nineteen studies were eligible for inclusion, encompassing 18 case series and one population-based study using national registry data. A total of 487 patients were included in the primary studies. The prevalence of ILNM in rectal cancer is 0.36%. ILNM are associated with very low rectal tumours with a mean distance from the anal verge of 1.1 cm (95% CI 0.92–1.27). Invasion of the dentate line was found in 76% of cases (95% CI 59–93). In patients with isolated inguinal lymph node metastases, modern chemoradiotherapy regimens in combination with surgical excision of inguinal nodes are associated with 5-year overall survival rates of 53–78%.

**Conclusion:**

In specific subsets of patients with ILNM, curative-intent treatment regimens are feasible, with oncological outcomes akin to those demonstrated in locally advanced rectal cancers.

**Supplementary Information:**

The online version contains supplementary material available at 10.1007/s10151-023-02826-x.

## Introduction

Inguinal lymph nodes are a rare but recognised site of metastasis in rectal adenocarcinoma. Lymphatic drainage of the rectum is primarily via the mesorectal nodes and subsequently via the nodal chains associated with the mesenteric vessels [1]. In very low rectal cancers, however, lymphatic spread has been demonstrated via inguinal nodes in a similar fashion to that seen in anal canal squamous cell cancers [2–5]. A further explanation for inguinal lymph node metastases (ILNM) has been hypothesised, whereby locally advanced disease obstructs the proximal mesorectal lymphatic pathway leading to alternate drainage via inferior superficial routes [6]. ILNM have been reported in both locally advanced disease and relatively early cancers, seemingly supporting the existence of both pathways [7–9].

At present, the American Joint Committee on Cancer (AJCC) considers ILNM as non-regional lymph node involvement. As such, ILNMs are deemed to represent distant metastatic or stage IV disease [10]. However, recent case series demonstrate significantly better survival outcomes for patients with ILNM treated with curative intent when compared to patients with distant solid organ metastatic disease [8, 9, 11], thereby suggesting the reduced survival outcomes associated with distant metastases should not apply to these patients.

No guidance exists for the management of ILNM in rectal adenocarcinoma. Furthermore, no accepted incidence rates or prognostic data are available beyond small case series. Therefore, there is limited evidence available to clinicians when treating patients with ILNM and treatment is primarily guided by the judgement and experience of local clinicians. This systematic review aims to collate and summarise all primary research involving ILNM from rectal adenocarcinoma to characterise patients with ILNM and ultimately provide clinicians with higher-level evidence for optimal management strategies.

## Methods

This systematic review has been reported according to the Preferred Reporting Items for Systematic Reviews and Meta-Analyses (PRISMA) guidelines [12]. A prospective review protocol was registered with the International Prospective Register of Systematic Reviews (PROSPERO) database (registration no. CRD42022385514).

### Search strategy

A systematic search was performed using PubMed, the Cochrane Central Register of Controlled Trials (CENTRAL) Library, Embase, Medical Literature Analysis and Retrieval System Online (MEDLINE) and Scopus databases. The following search algorithm, including exploded Medical Subject Headings (MeSH), was used: (Rectal cancer OR Rectal adenocarcinoma) AND (Inguinal lymph nodes OR inguinal lymphadenopathy OR inguinal lymph node metastases). Results were filtered to human studies published in the English language. The final searches took place in December 2022.

### Eligibility criteria

#### Inclusion

All randomised and non-randomised studies which report original data on the presentation, management or prognosis of patients with inguinal lymph node metastases associated with rectal adenocarcinomas were included.

#### Exclusion

Studies reporting on patients with anal squamous cell cancers were excluded from this review. All studies published in languages other than English were excluded, and studies available as conference abstracts only or those not published in peer-reviewed journals were excluded. Case reports were also excluded.

### Study selection and data extraction

Two authors independently screened each article identified by the initial search using the study title and abstract in reference to the eligibility criteria within the Rayyan software [13]. Conflicts were resolved via discussion. Screened articles were then included in a full-text review to confirm final eligibility. The database search was supplemented with forward and backward chaining of included study’s references and citations, in addition to utilising the “similar articles” feature within the PubMed database.

The primary outcome was treatment modality and subsequent survival. Secondary outcomes included prevalence, presentation and complications from treatment.

Data was manually and independently extracted onto a prospectively designed database. All case series underwent quality analysis using the critical appraisal tool developed by the Joanna Briggs Institute [14]. This tool is specific to case series and is designed to analyse the risk of selection, reporting and measurement bias.

### Statistical analysis

Pooled proportions were calculated using the pooled number of events and cases where possible. When summary statistics were reported in isolation, meta-analyses of means were calculated using a random-effects model and the metan command in Stata v14 (Stata Corp). Meta-analyses of means are presented with corresponding 95% confidence intervals. Survival rates were extracted from text or Kaplan–Meier curves [15]. When summary statistics were reported as a median, and a range or interquartile range (IQR), mean and standard deviation (SD) estimation was utilised [16, 17].

## Results

### Study selection

**Fig. 1:**
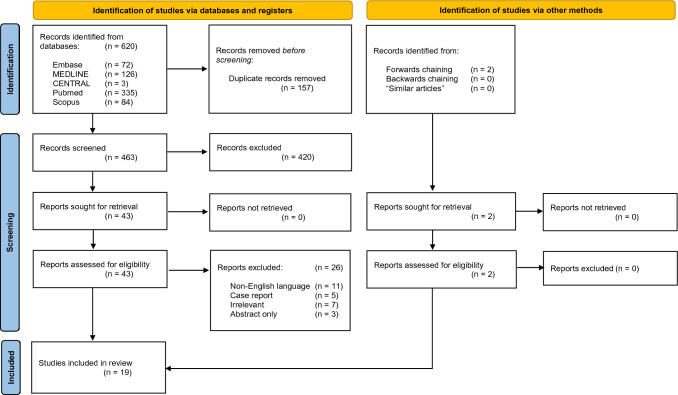
PRISMA diagram

The PRISMA diagram (Fig. 1) summarises the study selection process. The initial search yielded 620 references, from which 157 duplicates were removed. The remaining 463 records were screened down to 43 studies which underwent full-text review. Eleven studies reported in languages other than English [18–28], five case reports [29–33], seven irrelevant studies [4, 34–39] and three reports which were abstracts only [40–42] were excluded. Two further studies for inclusion were identified through forward chaining of citations. In total, 19 studies met the eligibility criteria and were included in the review [2, 8, 9, 11, 43–57].

### Study characteristics

The included studies comprise 18 case series: two are multicentre, and the remaining 16 are single-centre. One study is population-based and uses national registry data. In total, 487 patients with inguinal lymph node metastases from rectal adenocarcinoma are included. Time periods of patient recruitment in the included studies range widely, the earliest starting in 1949 and the latest included patients treated up to 2020. Table 1 summarises the study characteristics.

**Table 1:** Study characteristics

First author	Year published [reference]	Study period	Location	Site	Study design	Study *n*
Abd El Aziz	2022 [11]	2002–2020	USA	Single centre	Case series	24
Adachi	2013 [43]	1993–2010	Japan	Single centre	Case series	10
Bardia	2010 [44]	1995–2004	USA	Dual centre	Case series	6
Bebenek	2009 [2]	1998–2007	Poland	Single centre	Case series	6
Chen	2021 [45]	2017–2019	China	Single centre	Case series	16
Graham	1990 [46]	1949–1987	USA	Single centre	Case series	40
Hagemans	2019 [47]	2005–2017	Netherlands	Single centre	Case series	27
Hamano	2010 [48]	2000–2007	Japan	Single centre	Case series	7
Hasegawa	2022 [49]	2005–2019	Japan	Single centre	Case series	15
Luna-Perez	1999 [50]	1985–1996	Mexico	Single centre	Case series	32
Mesko	1994 [51]	1964–1990	USA	Single centre	Case series	18
Saiki	2022 [8]	1991–2006	Japan	Multicentre registry	Population-based study	40
Sato	2022 [9]	1997–2011	Japan	Multicentre	Case series	141
Shiratori	2020 [52]	2003–2019	Japan	Single centre	Case series	16
Tanabe	2019 [53]	1986–2017	Japan	Single centre	Case series	31
Tocchi	1999 [54]	1965–1990	Italy	Single centre	Case series	21
Ueta	2019 [55]	2005–2016	Japan	Single centre	Case series	7
Wang	2014 [56]	1986–2013	China	Single centre	Case series	20
Yeo	2014 [57]	2001–2011	Korea	Single centre	Case series	10

### Bias and quality analysis

Results of the bias and quality analysis are displayed in Table 2*.* Several studies demonstrated similar drawbacks, such as incomplete reporting of long-term outcomes and patient demographics. However, most studies scored highly for quality and presented sufficient data to limit bias.

**Table 2:** Risk of bias analysis

First author	Publication year [reference]	Were there clear criteria for the inclusion in the case series?	Was the condition measured in a standard, reliable way for all participants in the case series?	Were valid methods used for identification of the condition for all participants included in the case series?	Did the case series have consecutive inclusion of participants?	Did the case series have complete inclusion of participants?	Was there clear reporting of the demographics of the participants in the study?	Was there clear reporting of clinical information of the participants?	Were the outcomes or follow-up results of cases clearly reported?	Was there clear reporting of the presenting sites'/clinics' demographic information?	Was statistical analysis appropriate?
Abd El Aziz	2022 [11]	Y	Y	Y	Y	Y	Y	Y	Y	Y	Y
Adachi	2013 [43]	Y	Y	Y	N	N	Y	Y	Y	Y	Y
Bardia	2010 [44]	Y	Y	Y	Y	Y	Y	Y	Y	Y	Y
Bebenek	2009 [2]	Y	Y	Y	Y	Y	Y	Y	N	Y	Y
Chen	2021 [45]	Y	Y	Y	Y	Y	Y	Y	Y	Y	Y
Graham	1990 [46]	Y	Y	Y	Y	Y	N	N	N	Y	Y
Hagemans	2019 [47]	Y	Y	Y	Y	Y	Y	Y	Y	Y	Y
Hamano	2010 [48]	Y	Y	Y	N	N	Y	Y	Y	Y	Y
Hasegawa	2022 [49]	Y	Y	Y	Y	Y	Y	Y	Y	Y	Y
Luna-Perez	1999 [50]	Y	Y	Y	N	N	N	Y	N	Y	Y
Mesko	1994 [51]	Y	Y	Y	N	N	N	N	N	Y	Y
Sato	2022 [9]	Y	Y	Y	Y	Y	Y	Y	Y	Y	Y
Shiratori	2020 [52]	Y	Y	Y	Y	Y	Y	Y	Y	Y	Y
Tanabe	2019 [53]	Y	Y	Y	Y	Y	Y	Y	Y	Y	Y
Tocchi	1999 [54]	Y	Y	Y	Y	Y	N	N	N	Y	Y
Ueta	2019 [55]	Y	Y	Y	Y	Y	Y	Y	Y	Y	Y
Wang	2014 [56]	Y	Y	Y	Y	Y	Y	Y	N	Y	Y
Yeo	2014 [57]	Y	Y	Y	Y	Y	Y	Y	Y	Y	Y

*Y* yes, *N* no

### Patient and primary tumour characteristics

Table 3 summarises the characteristics of the included patients and primary rectal tumours. Rectal tumours tended to be very low, with a pooled mean distance from the anal verge of 1.1 cm, despite 20.8% of tumours being located more than 5 cm from the anal verge. Malignant invasion of the dentate line was common (76%), and tumours were more likely to be locally advanced (81.6% T3–T4 disease). However, involved mesorectal lymph nodes were not universal; 32% of patients had no mesorectal nodal involvement. Within the included patients, unilateral ILNM were more prevalent than bilateral ILNM (72.1% vs 27.9%).

**Table 3:** Characteristics of patients and primary rectal tumours

Patient or tumour characteristic/summary statistic	No. of studies reporting characteristic	No. of patients included in pooled average	Pooled average
Age (years), mean (95% CI)	16	409	62.4 (61.4–63.4)
Gender			
Male, *n* (%)	17	440	248 (56.4)
Female, *n* (%)			192 (43.6)
Height of primary rectal tumour			
0–5 cm from AV, *n* (%)	3	53	42 (79.2)
> 5–10 cm from AV, *n* (%)			9 (17)
> 10–15 cm from AV, *n* (%)			2 (3.8)
Distance from AV (cm), mean (95% CI)	7	111	1.10 (0.92–1.27)
Invasion of dentate line, % (95% CI)	6	98	76 (59–93)
Primary tumour staging			
T1, *n* (%)	10	136	4 (2.9)
T2, *n* (%)			21 (15.4)
T3, *n* (%)			54 (39.7)
T4, *n* (%)			57 (41.9)
N−, *n* (%)	12	181	58 (32.0)
N+, *n* (%)			123 (68.0)
Primary tumour differentiation			
Moderately/well differentiated, *n* (%)	8	140	96 (68.6)
Poor/mucinous, *n* (%)			44 (31.4)
Laterality of ILNM			
Unilateral, *n* (%)	13	383	276 (72.1)
Bilateral, *n* (%)			107 (27.9)
Presentation of ILNM			
Synchronous, *n* (%)	11	344	176 (51.2)
Metachronous, *n* (%)			168 (48.8)

*ILNM* inguinal lymph node metastases, *AV* anal verge, *CI* confidence interval

### Presentation

Just two studies report on the incidence of ILNM in rectal adenocarcinoma [8, 46]. When pooled, 80 patients from 22,130 cases of rectal cancer demonstrated ILNM, giving a pooled incidence of 0.36%. Within the included single-centre case series, a pooled mean of 17.8 cases was identified for every 10 years of the study periods. This figure can be extrapolated to suggest that large tertiary cancer centres see, on average, 1.8 cases of ILNM from rectal adenocarcinomas each year.

The most common definition of a metachronous presentation, as defined by primary studies, was the identification of ILNM within 1 year of the primary rectal tumour. Given this definition, synchronous ILNM had equal representation to metachronous presentations in the data set (51.2% vs 48.8%, respectively).

When reported, preoperative diagnosis of ILNM was with computed tomography (CT) and fluorodeoxyglucose positron emission tomography (FDG-PET). The most common definition of a clinically positive ILN was FDG-PET positivity, abnormal morphology or a short-axis diameter of 10 mm or greater [49, 52, 55]. Within the included studies, there is variable use of lymph node biopsy to histologically confirm metastases prior to ILN dissection, and no clear consensus is demonstrated [40, 44, 48, 53].

Out of 399 patients for whom it was reported, 196 (49.1%) were diagnosed with isolated ILNM metastases only, whilst 203 (51.9%) had distant solid organ metastases in addition to ILNM. Regarding synchronous ILNM only, 63.6% of patients had disease isolated to just the inguinal lymph nodes (isolated synchronous inguinal lymph node metastases, SILNM), and 36.4% had ILNM in addition to distant organ metastases (synchronous inguinal lymph node metastases and distant organ metastases, SILNM&DOM). For metachronous presentations, 24.5% were isolated (isolated metachronous inguinal lymph node metastases, MILNM), and 80.3% had distant metastases (metachronous inguinal lymph node metastases and distant organ metastases, MILNM&DOM). Given the expected differences in the treatment and prognosis of these four presentations, all outcomes were analysed in these four distinct groups.

### Treatment

#### Chemoradiotherapy

When reported, 95.1% (174 of 183) of patients from all groups had a radical rectal resection with curative intent. Eight of the remaining nine patients belonged to the SILNM&DOM group and were treated palliatively because of the presence of distant metastatic disease on presentation. The final patient demonstrated a complete clinical response of both the primary tumour and the ILNM to chemoradiotherapy and was successfully managed with a watch-and-wait approach.

The heterogeneity of the included studies limits meaningful analysis regarding the use of neoadjuvant chemoradiotherapy (NACR). The time period of patient recruitment and TNM staging vary widely. Accepting these limitations, pooled rates of NACR for isolated SILNM and SILNM&DOM are 65.2% and 92%, respectively. For isolated MILNM, 50% had NACR prior to rectal resection. For MILNM&DOM cases, reporting of NACR was insufficient to calculate summary rates.

External beam radiotherapy targeted at the inguinal nodes was sporadically reported upon. Therefore, mean rates for each group are not calculable. Abd El Aziz et al. report this in the greatest detail and use a similar dose (median 50.4 Gy, range 45–66) for both inguinal and rectum-targeted radiotherapy. The mean rate of use for all patients was 45.7%, but studies were polarised and reported high (79–100%) or low usage (0–3%), suggesting significant centre-specific variation in protocols.

#### Surgical treatment of ILNM

Ten studies reported on the surgical excision of ILNM, including 204 patients [9, 11, 40, 43, 45, 49, 50, 53, 55, 57]. Only two studies describe the surgical technique in detail [49, 53]. Both describe excisions which included both clinically positive and negative nodes. Abd El Aziz et al. [11] report the excision of clinically positive nodes only in some cases and the complete dissection of superficial inguinal lymph nodes in others. Furthermore, the depth of dissection was limited to the superficial inguinal nodes in two studies [40, 45], but Tanabe et al. and Hasegawa et al. describe additional dissection of the deep inguinal nodes [49, 53]. Abd El Aziz et al. also describe the use of minimally invasive lymph node dissection, an approach utilising laparoscopic ports within the femoral triangle [58, 59]. The remaining studies do not detail the technique. Given the available data, comparative analysis of long-term outcomes by surgical technique is not possible.

Three studies reported on postoperative complications of groin dissection for ILNM [47, 49, 55]. Clavien–Dindo [60] (CD) grade II and III complications were developed by 20.5% (8/39) and 17.9% (7/39) of cases, respectively. No CD grade IV or higher complications were reported. Complications included lymphorrhoea, seroma, wound infection and lymphoedema.

### Recurrence and survival

Heterogeneity and limited reporting in primary studies precluded comparative or proportional meta-analyses for recurrence or survival outcomes. Data synthesis is, therefore, descriptive.

Studies published before 2010 either did not report or reported poor survival outcomes for patients with any form of ILNM [2, 46, 50, 51, 54]. The largest study from this period, Graham et al. [46], considered treatment “predominantly palliative” given coexistent advanced pelvic or distant disease. Luna-Perez et al. [50] also report 0% 5-year overall survival and conclude that “only palliative treatment should be indicated”.

However, more recent studies have demonstrated improved outcomes, particularly for isolated ILNM. For isolated SILNM and MILNM, 1-, 3- and 5-year overall survival reported in the largest and most recent series are 82–100%, 53–86% and 53–78%, respectively [9, 11, 47, 49, 53]. Survival is worse for patients with concurrent distant organ metastases, whether this is found synchronously or metachronously. Abd El Aziz et al. included eight patients with SILNM&DOM who underwent NACR, curative intent resection and surgical excision of affected inguinal lymph nodes and reported 47% and 21% 3-year and 5-year overall survival, respectively. Yeo et al. report on seven patients with MILNM&DOM and demonstrate 0% 3- and 5-year survival with a mean overall survival of 14 ± 6.9 months from the time of recurrence.

When focusing on isolated SILNM, Hasegawa et al. and Abd El Aziz et al. report the highest 5-year overall survival for this cohort at 77.5% and 53%, respectively. All but one patient from both groups underwent neoadjuvant chemotherapy, and 25/29 (86.2%) underwent radiotherapy. Abd El Aziz et al. report radiotherapy was targeted rectally and at the inguinal region. Hasegawa et al. do not report on the targeted site of radiotherapy, but state all included patients underwent surgical excision of ILNM in addition to curative-intent radical rectal resection. Eight of 14 (57.1%) patients in the study by Abd El Aziz et al. had limited excision of clinically positive nodes, and the remainder from both studies had more extensive ILN dissection. Nine patients in each group had adjuvant chemotherapy. Within the patients reported on by Abd El Aziz et al., six patients developed distant recurrence, and two developed inguinal region recurrence. Both inguinal recurrences were contralateral to the operated side. For Hasegawa et al., six patients developed recurrence, four within the pelvis and two at distant sites. No inguinal recurrences were reported.

For isolated MILNM, no modern studies report survival and recurrence figures specific to this cohort. Three studies [11, 40, 53] report treating such patients with chemotherapy and ILN surgical dissection. One of these studies pooled survival rates with isolated SILNM and reported a 5-year overall survival of 55.3% for 31 patients [53]. More specific oncological outcome data for this cohort is not available in the current literature.

### ILNM response to chemoradiotherapy

Hasegawa et al. [49] performed PET-CT in all 15 patients in their series after NACR. All five patients without FDG uptake in ILN after NACR had no histopathological evidence of disease in the inguinal nodes retrieved during dissection. In the case of persistently FDG-avid PET-CT, just 4/9 (44.4%) were histologically positive for metastases. Within the isolated SILNM cohort, Abd El Aziz et al. reported 9/13 (69.2%) patients had histologically positive nodes after NACR.

## Discussion

This review demonstrates that for specific subsets of patients with ILNM, modern chemoradiotherapy regimens in conjunction with surgical ILN dissection can achieve survival rates significantly above that expected for rectal cancers with distant solid organ metastases [61]. In fact, for those with isolated SILNM, overall survival rates equal or surpass those published in large-volume trials of patients with rectal cancer with locoregional nodal involvement [62, 63].

To achieve higher survival rates, there appears to be a role for surgical dissection of the inguinal nodes. The centres demonstrating the best long-term oncological outcomes utilise a combination of NACR and surgical ILN dissection. From the data set, it is unclear whether dissection should be limited to clinically positive nodes or more extensive nodal clearance of the inguinal region. Abd El Aziz et al. demonstrated no ipsilateral inguinal recurrence after limited dissection of just clinically positive nodes in eight patients [11]. Both techniques have therefore been utilised and demonstrated success, but recommendations are not possible with such small sample sizes.

Furthermore, the optimal depth of dissection remains uncertain. Hagemans et al. suggest limiting dissection to superficial nodes only [47], a recommendation supported by their study of 17 patients who underwent superficial ILN dissection without inguinal recurrence. Hasegawa et al., however, suggest the additional excision of deep inguinal nodes [49]. Therefore, there is no consensus, and the limited data set precludes a recommendation.

Chemoradiotherapy appears to play an essential role. In two separate series reporting radiologically positive ILNM after NACR, just 4/15 (27%) and 9/13 (69%) had confirmed metastases on pathological examination, respectively [11, 49]. The limited data shows that inguinal nodes are likely responsive to systemic chemotherapy and radiotherapy. In Hasegawa et al.’s series, all patients without FDG uptake in ILN after chemoradiotherapy had negative histology, thereby suggesting that ILN dissection may not be necessary in such cases.

ILN dissection is associated with significant morbidity for patients. Wound complications such as infection, seroma, wound necrosis and lymphorrhoea occur in more than 50% of patients after ILN dissection for conditions such as melanoma or genital squamous cell cancers, where lymphadenectomy is performed more routinely [64, 65]. This review found wound complication rates of 38.5% specific to inguinal nodal clearance in cases of rectal adenocarcinoma. As such, ILN dissection should be employed with caution. This review finds 5-year overall survival rates of 0–21% for patients with distant solid organ metastases in addition to ILNM, not dissimilar to established survival rates for patients with stage IV disease [61, 66]. It would, therefore, appear harder to justify the morbidity of ILN dissection in such patients. However, if curative intent treatment is possible for the associated solid organ metastases, surgical excision may have a role.

ILNM are rare, as evidenced by the identification of under 500 eligible patients for this review. Almost all the primary data is found in case series of 40 patients or fewer. Selection and publication biases are, therefore, inherent risks. Additionally, reporting variability and inter-study heterogeneity are common, as demonstrated by each study’s highly variable inclusion criteria. The limitations in the primary data inevitably limit conclusions regarding the optimal treatment of ILNM. Conclusions are not, therefore, recommendations and are subject to challenge from original higher-quality evidence.

Further single-centre case series are unlikely to add significantly to the knowledge base. There is, therefore, a need for higher volume, impactful research in this field. Given the rarity of ILNM, standard study recruitment approaches would likely never reach the patient numbers sufficient for meaningful results. A multinational registry similar to the mASCARA registry for anal squamous cell cancers could provide additional data [67]. The inclusion of ILNM as a factor in existing registries for colorectal cancer should also be considered. Furthermore, expert consensus opinion would provide clinical teams treating patients with ILNM much-needed guidance. At present, however, this review represents a summation of the current understanding in this field and provides a contemporary overview for aid clinicians involved in treating patients with ILNM from rectal adenocarcinoma.

## Conclusions

Although rare, patients with inguinal lymph node metastases from rectal adenocarcinoma are likely to be encountered regularly in large centres. Optimal treatment strategies have not yet been established. Some centres have demonstrated successful management utilising chemoradiotherapy and surgical ILN dissection. For patients without accompanying distant solid organ metastases, such strategies demonstrate survival rates similar to patients with stage III disease. Therefore, ILNM can be considered as locoregional nodal involvement and treated accordingly.

### Supplementary Information

Below is the link to the electronic supplementary material.

Supplementary file1 (DOCX 39 KB)

## Data Availability

The datasets used and/or analysed during this review are available from the corresponding author on reasonable request.

## Abbreviations

ILNM: Inguinal lymph node metastases
MILNM: Metachronous inguinal lymph node metastases
MILNM&DOM: Metachronous inguinal lymph node metastases and distant organ metastases
NACR: Neoadjuvant chemoradiotherapy
SILNM: Synchronous inguinal lymph node metastases
SILNM&DOM: Synchronous inguinal lymph node metastases and distant organ metastases
